# Preimplantation death of xenomitochondrial mouse embryo harbouring bovine mitochondria

**DOI:** 10.1038/srep14512

**Published:** 2015-09-29

**Authors:** Manabu Kawahara, Shiori Koyama, Satomi Iimura, Wataru Yamazaki, Aiko Tanaka, Nanami Kohri, Keisuke Sasaki, Masashi Takahashi

**Affiliations:** 1 Laboratory of Animal Breeding and Reproduction, Research Faculty of Agriculture, Hokkaido University, Kita-ku Kita 9 Nishi 9, Sapporo 060-8589, Japan

## Abstract

Mitochondria, cellular organelles playing essential roles in eukaryotic cell metabolism, are thought to have evolved from bacteria. The organization of mtDNA is remarkably uniform across species, reflecting its vital and conserved role in oxidative phosphorylation (OXPHOS). Our objectives were to evaluate the compatibility of xenogeneic mitochondria in the development of preimplantation embryos in mammals. Mouse embryos harbouring bovine mitochondria (mtB-M embryos) were prepared by the cell-fusion technique employing the haemagglutinating virus of Japan (HVJ). The mtB-M embryos showed developmental delay at embryonic days (E) 3.5 after insemination. Furthermore, none of the mtB-M embryos could implant into the maternal uterus after embryo transfer, whereas control mouse embryos into which mitochondria from another mouse had been transferred developed as well as did non-manipulated embryos. When we performed quantitative PCR (qPCR) of mouse and bovine *ND5,* we found that the mtB-M embryos contained 8.3% of bovine mitochondria at the blastocyst stage. Thus, contamination with mitochondria from another species induces embryonic lethality prior to implantation into the maternal uterus. The heteroplasmic state of these xenogeneic mitochondria could have detrimental effects on preimplantation development, leading to preservation of species-specific mitochondrial integrity in mammals.

Mitochondrial functions in the cell vary widely, and include ATP synthesis, metabolic integration, reactive oxygen species synthesis, and the regulation of apoptosis^1^. Among these, ATP synthesis through oxidative phosphorylation (OXPHOS) provides almost all the energy required by eukaryotic cells. Mitochondrial DNA (mtDNA) is independent of nuclear DNA (nDNA), and the uniparental, maternal inheritance of mtDNA has been addressed in previous animal studies^2^.

The organization of mtDNA is remarkably uniform across species, reflecting its vital role in OXPHOS. Thus, the characteristic features of animal mtDNA are thought to have evolved after the divergence of the multicellular ancestors from the unicellular progenitors^3^. In mammals, mtDNA is about 16 kilobase pairs in length (e.g., cattle: 16338 bp [GenBank ID: NC_006853]; mouse: 16299 bp [GenBank ID: NC_005089]), and consists of a closed circular double-stranded DNA that encodes the 13 essential subunit proteins of the OXPHOS, two ribosomal RNAs, and the 22 transfer RNAs required for mitochondrial protein synthesis^4^. Therefore, mtDNA has been used extensively in mammalian phylogenetic studies^5^,^6^,^7^,^8^,^9^,^10^,^11^.

There is no question that mitochondrion is essential for complex multicellular organisms. Mitochondrial dysfunction results in a wide range of metabolic and degenerative diseases, and even aging in humans^12^,^13^. MtDNA is rigorously uniparentally (maternally) inherited, because sperm mitochondria are ubiquitinated in the ooplasm after fertilization and are subsequently proteolyzed during preimplantation development^14^. The homoplasmy that arises from uniparental maternal mtDNA inheritance can be changed experimentally to a heteroplasmic state by oocyte/egg cytoplasmic transfer (CT), in which oocyte cytoplasm containing mitochondria is transferred into another oocyte by microinjection or electrofusion, to study nDNA and mtDNA interactions^15^,^16^,^17^.

Intrasubspecies and intrafamily CT in mice (NZB/BinJ ↔ BALB/cByJ) and cattle (buffalo [*Bubalus bubalis*] ↔ cattle [*Bos taurus*]) allowed full-term development of cytoplasmic-hybrid embryos^17^,^18^. Furthermore, intragenus CT embryos in cattle (*Bos taurus* ↔ *Bos indicus*) resulted in the birth of a calf with a heteroplasmic mitochondrial state^15^. These studies suggest that the presence of cytoplasm from foreign oocytes/eggs, even from relatively close species, never prevent heteroplasmic embryos from developing to term. However, in a previous study, no hybrid mouse embryos constructed by interspecies CT of activated pig oocyte cytoplasm resulted in live offspring, although those authors presumed that mitochondrial heteroplasmy might not be the cause of the developmental failure^19^. Thus, it remains controversial to what extent the coexistence of mitochondria, albeit evolutionally conserved, from different species in an embryo affects mammalian ontogeny.

Most early studies transferred donor cytoplasm from intrinsic oocytes/embryos into a recipient embryo, but this method could not exclude the plausible effects from organelles other than mitochondria. Here, we produced xenomitochondrial mouse embryos harbouring bovine mitochondria (mtB-M embryos), using a centrifugation technique to collect mitochondria-enriched fractions, as reported previously^15^,^20^. We first assessed the localization of the bovine mitochondria in the mtB-M embryos during the preimplantation stage by using a fluorescence labelling reagent, Mito Tracker green^21^. Next, we determined to what developmental stage the mtB-M embryos could develop after embryo transfer into pseudopregnant females, in comparison with mouse–mouse CT control embryos (mtM-M embryos). Additionally, we evaluated the mtDNA content of mtB-M embryos by quantitative PCR, in which we used bovine- and mouse-specific primers for *ND5*, respectively. We found that interspecies CT of mitochondria harbouring markedly conserved genetic material was incompatible with life, which may be because genomic evolution of both nDNA and mtDNA had progressed uniquely in each mammalian species since the establishment of eukaryotic cells.

## Results

### Localization of bovine mitochondria transferred into mouse embryos

**Figure 1:**
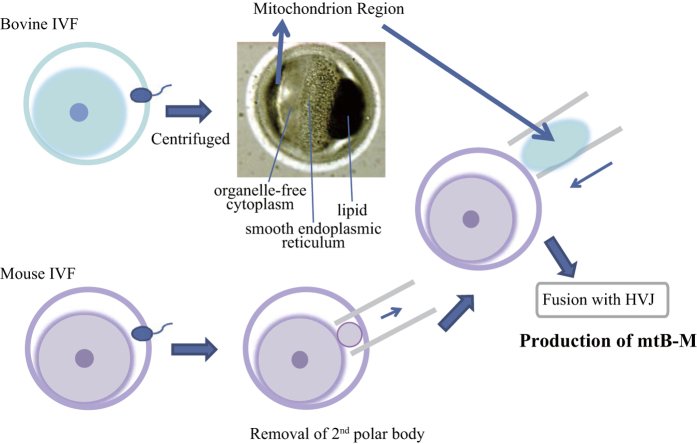
Construction of mouse embryos harbouring bovine mitochondria. After bovine IVF embryos were centrifuged, the zona pellucida on the side of the mitochondria- enriched domain was cut. In mouse embryos, the zona pellucida was cut near the second polar body in order to remove the second polar body. The bovine mitochondria-enriched domain was suctioned into a glass pipette using a microinjector, followed by drawing up a small volume of the inactivated HVJ solution. The suctioned cytoplasm of the bovine mitochondria-enriched domain including the HVJ solution was inserted into the perivitelline space of mouse embryo.

The bovine mitochondria-enriched regions were prepared by centrifugation of bovine *in vitro* fertilization (IVF) embryos, which were subsequently transferred into the perivitelline space of mouse IVF embryos after removal of their second polar bodies by micromanipulation (Fig. 1). After inducing cell fusion by means of the haemagglutinating virus of Japan (HVJ), fused mtB-M embryos were cultured to the blastocyst stage *in vitro*.

**Figure 2:**
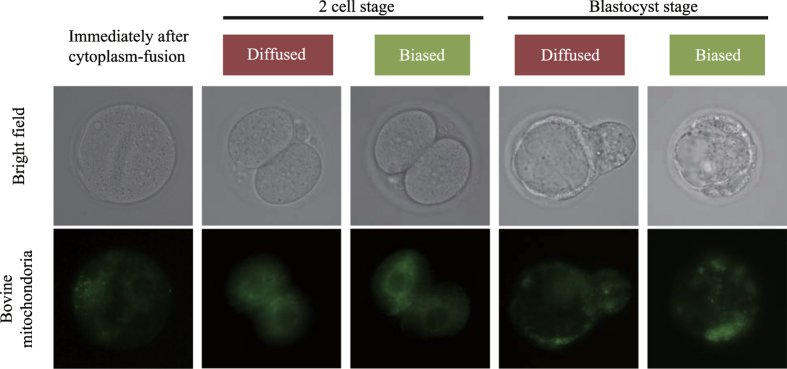
Localization of bovine mitochondria transferred into mouse embryos. Prior to micromanipulation, bovine mitochondria were labelled with Mito Tracker green, as shown in the lower row. Fluorescent signals showing bovine mitochondria in the mtB-M embryos were monitored at the stage immediately following cell fusion, the 2-cell stage, and the blastocyst stage. There were two types of staining pattern; namely, biased and uniform staining patterns at each developmental stage.

An experiment was designed to investigate how bovine mitochondria transferred into mouse embryos localized within the mtB-M embryos during preimplantation development (Fig. 2). After obtaining bovine mitochondria-enriched fractions by means of centrifugation, the embryos were stained with Mito Tracker green. Immunolabelling revealed that the transferred bovine mitochondria localized within the mouse embryos with either a restricted or uniform distribution at the two-cell and blastocyst stages (Fig. 2). These localization patterns may be due to the time lag from completion of cytoplasm fusion to the first cleavage, because the transferred bovine mitochondria were restricted to a plausible fusion region immediately after cytoplasm fusion. After the two-cell stage, if bovine mitochondria were restricted to a unilateral location in the blastomere, the bovine mitochondria in the mtB-M blastocysts were also polarized in a single direction. Thus, we showed that bovine mitochondria were present in the mtB-M embryos during preimplantation development, with either a restricted or uniform distribution. Although most probes such as Mito Tracker are toxic in general, the small volume of introduced bovine mitochondria might permit the mtB-M embryos to develop to the blastocyst stage.

### Effect of the presence of bovine mitochondria on mouse embryo development *in vitro* and *in vivo*

We designed a second experiment to determine the development potential of mtB-M embryos *in vitro* and *in vivo*. As a developmental control, we prepared mouse embryos (mtM-M) in which mitochondria from another mouse embryo were transferred in the same way used to produce mtB-M embryos. At first, we investigated the *in vitro* rate of development of both mtB-M and mtM-M embryos to the blastocyst stage (Table 1). The mtB-M embryos showed a significantly decreased blastocyst development rate (26.3% ± 2.7%) at E3.5, compared to those of the mtM-M and non-manipulated IVF embryos (92.7% ± 1.2% and 93.0% ± 2.9%, respectively). At the first cleavage, there were no significant differences in the rates of development for two-cell stage embryos among the experiment groups. However, both the mtM-M and non-manipulated IVF embryos reached the blastocyst stage at E3.5, while some mtB-M blastocysts only formed at E4.5 (10.6 ± 4.2%). This retardation of development suggested that a xenomitochondrial heteroplasmic state has detrimental effects on preimplantation development.

To clarify this possibility, we further performed embryo transfer of mtB-M embryos into pseudopregnant females and determined the potential for these embryos to develop to the postimplantation stage until E12.5 (Table 2). Non-manipulated IVF and mtM-M embryos showed normal foetal development at E12.5 (39.5 ± 11.7% and 26.7 ± 3.3%, respectively), whereas no mtB-M embryos developed to E12.5, and did not even implant. These findings clearly demonstrated that the mtB-M embryos not only demonstrated a delay in development from the two-cell stage to the blastocyst stage, but also showed preimplantation lethality.

### Assessment of the bovine mitochondria content in xenomitochondrial mouse embryos

**Figure 3:**
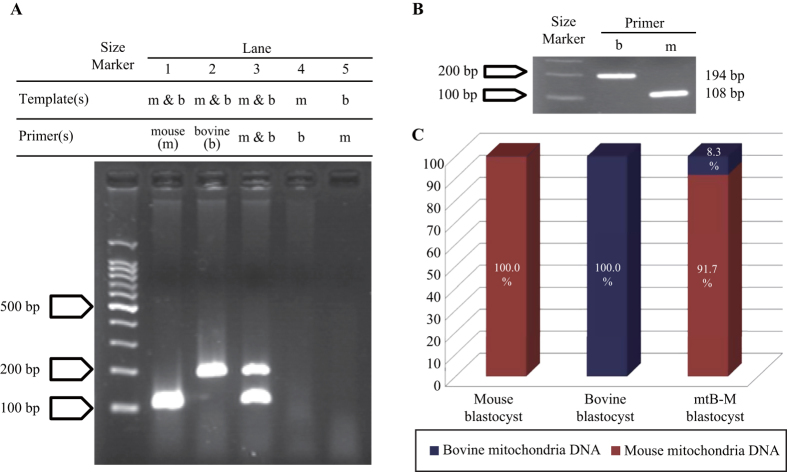
Bovine mitochondria content in xenomitochondrial mouse embryo. A third experiment was conducted to determine the bovine mtDNA content that can induce developmental delay and preimplantation lethality. (**A**) The species-specificity of primers for *ND5* in mouse (m) and cattle (**b**) was ensured by performing RT-PCR using three different types of mitochondrial (mt)DNA templates: mouse tail-derived mtDNA, bovine oviduct-derived mtDNA, and a mixture (m & b) of these mtDNAs. Each primer set allowed us to detect species-specific amplicons of mouse and bovine *ND5*, 108 bp and 194 bp in length, respectively. (**B**) Using mtDNA from the mtB-M blastocysts, RT-PCR for *ND5* was performed using the same procedure, and species-specific amplification was verified (mouse: 108 bp; bovine: 194 bp). (**C**) Using these same primer sets, mouse and bovine mtDNAs were quantified in mouse (left), bovine (middle), and mtB-M (right) embryos using qPCR. Red and dark blue in the bar graph indicate the proportion of mouse and bovine mtDNA within a blastocyst, respectively. Independent experiments were repeated three times. Standard deviations were 0.00276, 0.00002, and 2.95761 in mouse, bovine, and mtB-M embryos, respectively.

A third experiment was conducted to determine the effect of bovine mtDNA content on induction of developmental delay and preimplantation lethality. We first designed species-specific PCR primer sets for mouse and cattle *ND5* (encoding NADH dehydrogenase 5). As shown in Fig. 3A, the species-specificity of the primers was verified by performing RT-PCR using the following three types of total DNA templates: mouse (m) tail-derived total DNA, bovine (b) oviduct-derived total DNA, and a mixture of these total DNA (m and b; Fig. 3). The primer sets allowed us to detect species-specific PCR products of mouse and bovine *ND5*, which were 108 bp and 194 bp in length, respectively (Fig. 3A). Because the mtB-M embryos also exhibited fragments of these two sizes, we confirmed the presence of both bovine and mouse mtDNA (Fig. 3B). Using the same primer sets, we quantified both bovine and mouse mtDNA in mtB-M embryos using quantitative RT-PCR (Fig. 3C). We determined that the mtB-M embryos possessed 8.3% bovine mitochondria. These results demonstrated that contamination with less than 10% of bovine mitochondria causes a delay in development as well as preimplantation lethality in mouse embryos.

### Integrity of isolated bovine mitochondria

**Figure 4:**
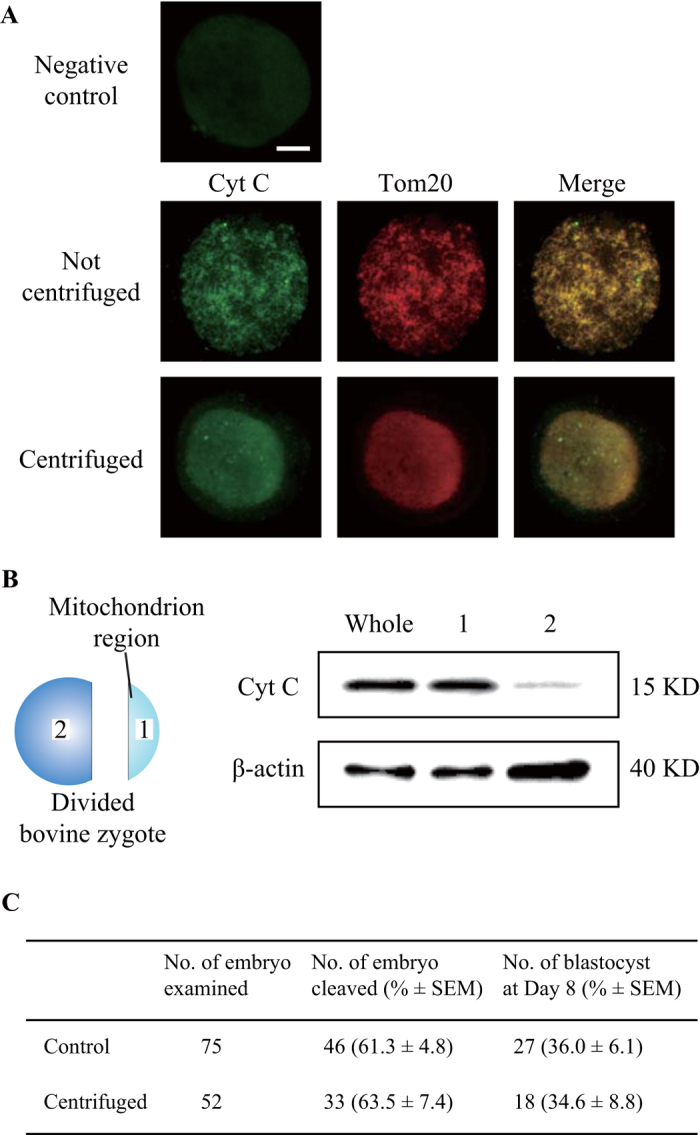
Cytochrome c protein expression in centrifuged bovine embryo. In order to validate the integrity of the bovine mitochondria after centrifugation, we examined the release of cytochrome c protein with the localization of Tom20 that is a subunit of the translocase of the outer membrane (TOM) complex. (**A**) Fluorescence micrograph showing distribution of cytochrome c and Tom20 staining in not-centrifuged and centrifuged bovine embryos. The top image was background fluorescence signals in the negative control lacking the primary antibody treatment. The fluorescent intensity in another cytosolic fraction in centrifuged embryo was similar to that of the negative control. Bar = 50 μm. (**B**) Comparison of cytochrome c protein expression between mitochondria-enriched fraction (1 in the left illustration) and another cytosolic fraction (2) by WB. Whole: whole embryo; 1: mitochondria-enriched fraction; 2: another cytosolic fraction. (**C**) Effect of centrifugation treatment on the development into the blastocyst stage in bovine embryo.

In order to validate the integrity of the bovine mitochondria after centrifugation, we examined the release of cytochrome c protein in bovine mitochondria. When the embryos are stressed, cytochrome c is released from their mitochondria^22^. Therefore, to detect whether cytochrome c was released from mitochondria as a marker of mitochondrial-mediated apoptotic events, we examined cytochrome c protein in centrifuged bovine embryos by immunohistochemistry and western blotting analyses. Immunostaining (IC) showed that the negative control, lacking the first antibody treatment, displayed only the nonspecific signals of secondary antibody (Fig. 4A). In the centrifuged bovine embryo, on the right side in this figure, the mitochondria-enriched domain showed intense signals, whereas the left side showed dilute signals, similar to the negative control. We next compared cytochrome c protein expression between the mitochondria-enriched fraction and another cytosolic fraction obtained during mitochondrial isolation by western blotting. This revealed that cytochrome c protein expression in the mitochondria-enriched cytosolic fraction was similar to that in the whole embryo (Fig. 4B). As expected, cytochrome c protein expression in another cytosolic fraction was rare. To further validate bovine mitochondrial integrity in the centrifuged embryos, we compared the development until the blastocyst stage between non-centrifuged and centrifuged IVF embryos. Centrifuged embryos clearly exhibited the same developmental competence as the non-centrifuged controls (Fig. 4C). Thus, the mitochondrial integrity after isolation is maintained in bovine embryos. This result is consistent with previous work using centrifuged oocytes/embryos from other groups^23^,^24^,^25^.

## Discussion

In this study of mouse embryos containing bovine mitochondria, we have clearly demonstrated that a xenomitochondrial heteroplasmic state blocks embryonic development at the post-implantation stage in mammals. Introduction of bovine mitochondria into mouse embryos reduced the number of embryos that reached the blastocyst stage at the expected time point, and none of these even resulted in formation of implantation sites in the recipient uterus after embryo transfer. This block of embryonic development was induced by contamination with xenogeneic mitochondria of less than 10%. The developmental arrest did not depend on the localization pattern of the introduced xenogeneic mitochondria.

The mechanism underlying the inhibition of development by xenomitochondrial heteroplasmy is unknown. One possibility is that a xenomitochondrial heteroplasmic state induces a delay of development up to the blastocyst stage, which may be due to impaired mitochondrial function, causing an increase in the percentage of delayed embryos^26^,^27^. It is known that impairment of mitochondrial function by treatment with inhibitors that interrupt mitochondrial metabolic function disturbs normal foetal and placental development in the mouse embryo^26^.

In an earlier study, the development of pig–mouse cytoplasmic hybrid embryos was evaluated to address the nuclear-cytoplasmic incompatibility of interspecies somatic cell nuclear transfer embryos. The present study further focused on the role of mitochondria by transferring mitochondria-enriched cytoplasm. We found that when the mtB-M embryos were transferred to pseudopregnant females, they failed to develop by E12.5, and could not even form implantation sites. These results demonstrated that xenomitochondrial contamination induces preimplantation death of the mtB-M embryos. Moreover, the mtB-M embryos were able to develop into blastocysts, although the rate of development was slower than that of the non-manipulated IVF and mtM-M embryos. The pattern of localization of bovine mitochondria by the blastocyst stage varied, and is therefore unlikely to have an impact on the developmental potential of the mtB-M embryos. Thus, our results indicated that the exogenous interspecies mitochondrial contamination per se impairs the viability of the embryo.

Mitochondrial dysfunction is associated with a surprisingly broad range of clinical phenotypes^28^. Because, mitochondria play a pivotal role in energy production in the form of ATP by the process of OXPHOS, or aerobic respiration, cells carrying mtDNA with pathogenic mutations result in severe metabolic disorders, characterized by aberrant cellular respiration^29^,^30^,^31^,^32^. Furthermore, mice carrying respiration-deficient mitochondria with deletion of six tRNA genes and seven structural genes were generated, termed mito-mice, which showed a variety of anomalies, including aberrant muscle fibres, hyperplastic kidneys, and high concentrations of lactic acid and pyruvate in the peripheral blood^33^,^34^. Importantly, the pathological phenotypes of the mito-mice were largely dependent on the proportions of mutant mtDNA^33^,^34^. When abnormal mitochondria accumulated to a level of more than approximately 80% of all the mitochondria in a tissue, pathogenic phenotypes manifested as a result of mitochondrial dysfunctions. Likewise, the mtB-M embryos are mouse embryos harbouring exogenous bovine mitochondria, in a plausible mosaic state, as determined by observation of Mito Tracker staining (Figs 1 and 3). However, although the proportion of bovine mitochondria in the mtB-M embryos (8.3%, as shown in Fig. 3) was much less than 80%, none of the mtB-M blastocysts could develop up to E12.5, or even implant into the maternal uterus (Table 2). This severe lethality of the mtB-M embryos was clearly due to contamination with bovine mitochondria. Isologous cytoplasm hybrid embryos, mtM-M embryos, could develop into the blastocyst stage at the normal speed, and reached foetal stage by E12.5, as did the non-manipulated IVF embryos.

When involving closely related species, a xenomitochondrial heteroplasmic state does not impair cell viability. Human mtDNA-free cells harbouring mtDNA from chimpanzee and gorilla showed a functional OXPHOS system and were viable^35^. Mouse mitochondrial DNA-free cells containing rat mitochondrial DNA were also viable, but were defective in respiration^36^. The bovine mitochondria introduced into the mouse embryos were not eliminated, at least until the blastocyst stage, whereas none of the mtB-M embryos developed until E12.5, without formation of implantation sites. Mitochondria can interact through transport of DNA products, which is thought to be essential for the defensive mechanism of mitochondrial function in cells^37^. After development into the blastocyst stage, DNA products from the introduced bovine mitochondria may thus have impaired mouse mitochondrial functions, and consequently, may have had detrimental effects on embryo development.

Mouse embryos can develop and subsequently implant in the absence of mitochondrial respiration, although the development of such embryos does not go to term^38^,^39^,^40^. Up to the blastocyst stage, aerobic respiration via mitochondria is upregulated with the compaction of mitochondrial cristae, resulting in the utilization of glucose as the carbohydrate substrate increases^41^,^42^. Therefore, the metabolism mediated by the mitochondria in an early embryo is immature up to the blastocyst stage, after which this metabolism begins to mature. In humans, mitochondria within oocytes and early stage embryos have few matrix membrane folds and are relatively inactive^43^,^44^. However, surprisingly, mouse embryos contaminated with only 8.3% bovine mitochondria did not implant, even though those embryos possessed intact mouse mitochondria at a level sufficient for cell survival. Mitochondrial function is dependent on the integrity of both the mtDNA and the nDNA, in terms of the transcription and translation machinery that converts their genetic information into proteins^13^,^45^. Incompatibility between bovine mitochondrial- and mouse nuclear-encoded gene transcripts in the mtB-M embryos may be responsible for the observed preimplantation lethality.

Most mtDNA structures are extremely conserved in mammals, although mammalian species adapt to environments that differ from their original habitats^5^. Therefore, the protection of their own genetic material from invasion by that of other species would be required for preserving the identity of the species. A precise interdependence between nDNA and mtDNA is essential for the coordination of cellular signalling and transcription, to ensure that mitochondrial function is maintained in a species-specific manner^35^,^46^. However, little is known about how species-specific regulation of nuclear–cytoplasmic mutuality is maintained during mammalian development. Investigation of development in xenomitochondrial embryos would provide a good model for understanding the boundary of nuclear–cytoplasmic interdependence between species.

## Methods

### Ethical approval

All the animal experiments in this study were approved by the Institutional Animal Care and Use Committee, Hokkaido University, and were performed in accordance with National University Corporation Hokkaido University Regulations on Animal Experimentation.

### Chemicals

All chemicals were obtained from Sigma–Aldrich (St. Louis, MO, USA) unless otherwise stated.

### Preparation of bovine and mouse embryos

Bovine embryos were produced by IVF from oocytes obtained from abattoir-collected ovaries. All procedures for bovine IVF embryo production followed techniques previously described^47^,^48^. At 6 h after insemination, presumptive bovine zygotes were transferred into 100 μL of mSOFaa culture medium^48^, and were subsequently freed from cumulus cells and spermatozoa by pipetting gently. Before CT, mitochondria-enriched regions of bovine embryos were prepared by centrifugation for 15 min at 35 °C, at 10000 × *g*, in the culture medium that was supplemented with 7.5 μg/mL cytochalasin B, as reported in a previous study^20^, and these were incubated in the culture medium until required for use. In the experiment to investigate the effect of centrifugation treatment on the development into the blastocyst stage, centrifuged embryos were washed three times with mSOFaa and cultured for *in vitro* development to the blastocyst stage until day 8.

Mouse embryos were also prepared by IVF according to a previously described procedure^49^. Briefly, female ICR mice were superovulated by injections of 5 IU eCG (ASKA Pharmaceutical Co., Ltd., Tokyo, Japan) and 5 IU hCG (ASKA Pharmaceutical Co., Ltd.), given 48 h apart. Oocytes at metaphase II were released from the murine oviducts at 14 h after hCG administration. Sperm were collected from the cauda epididymis of mature male ICR mice and suspended in a 100-μL drop of T6 medium in paraffin oil^50^, and preincubated for 1 h in an atmosphere of 5% CO_2_ at 37 °C. The collected oocytes were transferred into a 100-μL drop of T6 medium in which the sperm concentration was adjusted to 0.5–1 × 10^6^ cells/mL. At 6 h after insemination, the embryos were denuded of cumulus cells by gentle pipetting in 0.5% hyaluronidase solution, washed with M2 medium, and transferred into a drop of M16 medium. The mouse embryos were incubated in M16 medium until used for CT^50^.

### Construction of mitochondrial hybrid mouse embryos harbouring bovine mitochondria

All manipulation procedures were performed after modification of the nuclear transfer protocol for the bi-maternal mouse embryos^51^ as necessary for CT. Mouse embryos harbouring bovine mitochondria were constructed by micromanipulation using an inverted microscope (Nikon, Tokyo, Japan) equipped with a set of micromanipulators including microinjectors (Narishige, Tokyo, Japan). The zona pellucida of both bovine and mouse embryos were cut with a glass knife before CT in M2 medium supplemented with 7.5 μg/mL cytochalasin B and 0.1 μg/mL colcemid (Wako Pure Chemical Industries, Ltd., Osaka, Japan). In bovine embryos after centrifugation, the zona pellucida on the side of the mitochondrial-enrichment region was cut. In mouse embryos, the zona pellucida near the second polar body was cut for the removal of the second polar body, using a 30-μm (external diameter) glass pipette. The bovine mitochondrial-enrichment region was suctioned into a glass pipette using a microinjector (Narishige); a small volume of inactivated haemagglutinating virus of Japan (HVJ; Ishihara Sangyo Kaishya, Ltd., Osaka, Japan) solution was then also drawn up into the pipette. The bovine mitochondrial-enrichment region and HVJ solution was then inserted into the perivitelline space of a mouse embryo lacking the second polar body. The amount of cytoplasm was limited within the perivitelline space of mouse embryo. These mouse embryos were then transferred into a 100-μL M16 droplet, and incubated at 37 °C for 30–60 min in an atmosphere of 5% CO_2_. After cell fusion was confirmed under a stereomicroscope, the mtB-M embryos were transferred into 50-mL M16 droplets, and were subsequently cultured and their development examined *in vitro* and *in vivo*.

To assess the *in vitro* development of these embryos into the blastocyst stage, the mtB-M embryos were cultured to E3.5. To investigate the delay in development of the mtB-M embryos, the embryos were cultured for another day, until E4.5. The mtB-M embryos that developed to the blastocyst stage at E3.5 were transferred into the uterine horns of recipient female mice at 2.5 days of pseudopregnancy, to investigate the postimplantation developmental potential. Autopsies to assess postimplantation development were carried out at E12.5, by which time the mouse foetus has completed the major organogenesis, including development of the placenta after implantation site formation.

### Sub-cellular localization of bovine mitochondria transferred into mouse embryos

To investigate the integrity and the sub-cellular localization of bovine mitochondria within the mtB-M embryos, bovine mitochondria were labelled with 50 nM Mito Tracker green (Life Technologies, Carlsbad, CA, USA) before micromanipulation^21^. The mtB-M embryos, which had been stained by incubation in mSOFaa medium containing Mito Tracker green for 30 min, were observed at the stage immediately after cell fusion, the two-cell stage, and the blastocyst stage, under a fluorescence microscope (KEYENCE BZ-9000, Osaka, Japan).

### Design of species-specific PCR primer sets for mouse and bovine mtDNAs

Prior to the assessment of the heteroplasmy level in the mtB-M blastocysts, we confirmed species-specific PCR amplification. The primer sets were designed for bovine *ND5* (b*ND5*; F: 5′-TACGGACGAGCAGATGCAAA-3′; R: 5′-TTTCCGGTTGCAGCTAATGC-3′; GenBank ID: NC_006853; location: 12583-12776; size: 194 bp) and mouse *ND5* (m*ND5*; F: 5′-TGCCTAGTAATCGGAAGCCTCGC-3′; R: 5′-TCAGGCGTTGGTGTTGCAGG-3′, GenBank ID: NC_005089; location: 12855–12962; size: 108 bp), respectively. To examine species-specificity of amplification, total DNA was isolated from abattoir-collected bovine oviducts and from mouse tails. The total DNA, which includes mtDNA, was used for PCR amplification with GoTaq DNA polymerase (Promega, Madison, WI, USA), 5 mM of each of the forward and reverse primers, and 1.0 mM of each dNTP. The cycling profile included an initial denaturing step for 5 min at 95 °C, followed by 35 cycles each consisting of 94 °C for 0.5 min, 62 °C for 1 min, and 72 °C for 1 min, and a final extension step at 72 °C for 2.5 min. The combination of the primer sets and templates used is shown above the result of electrophoresis analysis in Fig. 3A. The mouse and bovine mtDNA mixture was prepared by mixing equal amounts of the respective total DNAs.

### Quantification of the mtDNA content of blastocysts

After confirmation of species-specific PCR amplification using tissue samples, we next examined the mtDNA content of blastocysts with a qPCR assay for an *ND5* sequence. Total DNA was isolated from blastocysts using a QIAamp DNA Micro Kit (Qiagen, Valencia, CA, USA) according to the protocol provided by the manufacturer. The proportions of mouse and bovine mtDNA were determined by qPCR using each species-specific primer set on a LightCycler 480 (Roche Diagnostics, Basel, Switzerland). We first constructed standard curves for mouse and bovine *ND5* dilution series, ranging from 10^4^ to 10^7^ copies/μL, which were then used as external standards for determining mtDNA copy number. As a preliminary examination, we had confirmed that the mtDNA copy number of a mouse or bovine blastocyst amounted to about 10^5^ to 10^7^ copies. The quantification assay was replicated three times.

### Immunohistochemistry for localization of cytochrome c

Centrifuged bovine embryos were fixed with 4% paraformaldehyde in Dulbecco’s phosphate-buffered saline containing 0.02% polyvinyl alcohol (PBS/PVA) for 1 h at room temperature. The embryos were incubated in PBS containing 3% foetal bovine serum for 1 h at room temperature, and then were incubated overnight at 4 °C with mouse monoclonal anti-cytochrome c antibody (1:400; Abcam, Cambridge, UK). After washing with PBS/PVA, the embryos were incubated with Alexa Fluor 488-conjugated anti-mouse IgG secondary antibody (1:300; Life Technologies) for 1 h at room temperature. We further performed immunostaining for Tom 20 as a mitochondrial marker^52^. According to the procedure of cytochrome c immunostaining, rabbit polyclonal anti-Tom 20 antibody (1:200; Santa Cruz Biotechnology, CA, USA) and Alexa Fluor 555-conjugated anti-goat IgG secondary antibody (1:400; Life Technologies) were used. After four-times wash with PBS/PVA, embryos were mounted on glass slides. Images were obtained using a fluorescence microscope (KEYENCE). For the preparation of negative controls, the procedure was performed using bovine embryos in the absence of primary antibody treatment and centrifugation.

### Detection of cytochrome c protein by western blotting

The three types of embryo samples were prepared for western blotting by micromanipulation as described above; namely, mitochondria- enriched fractions, another cytosolic fractions obtained during mitochondrial isolation, and whole embryos without centrifugation. Proteins in lysates of 40 pooled embryo samples were electrophoresed using a 15% SDS-polyacrylamide gel and transferred to polyvinylidene difluoride membranes (Bio-Rad, Hercules, CA, USA). The membranes were blocked for 1 h with PBS containing 4% nonfat dried milk and 0.1% Tween-20, and then incubated overnight at 4 °C with primary antibodies (cytochrome c; 1:4000 and anti-β-actin; 1:20000) diluted in PBS containing 2.5% BSA. Subsequently, the membranes were washed in PBS containing 0.05% Tween 20 and incubated for 45 min at room temperature with the appropriate secondary horseradish peroxidase conjugated antibodies diluted in PBS containing 3% nonfat dried milk and 0.05% Tween 20 (1:5000). The immunoreactive bands were detected using Luminate Forte Western HRP substrate (Millipore, Billerica, MA, USA). The images of the protein bands were obtained with a Bio-Rad ChemiDoc™ EQ densitometer (Bio-Rad).

### Statistical analysis

Data were statistically analysed using one-way analysis of variance (ANOVA) and the Fisher PLSD test, using Statview software (Abacus Concepts, Inc., Berkeley, CA, USA). A *P*-value of < 0.05 was considered significant.

## Additional Information

**How to cite this article**: Kawahara, M. *et al.* Preimplantation death of xenomitochondrial mouse embryo harbouring bovine mitochondria. *Sci. Rep.* **5**, 14512; doi: 10.1038/srep14512 (2015).

## Figures and Tables

**Table 1:** Development of the mouse embryos harbouring bovine mitochondria into the blastocyst stage.

	No. of embryos examined	No. of embryos cleaved(% ± SEM)	No. of blastocysts at E3.5(% ± SEM)	No. of blastocysts at E4.5(% ± SEM)
non-manipulated	89	86 (96.6 ± 1.4)	80 (93.0 ± 2.9)	NE
mtM-M	57	55 (96.5 ± 2.6)	51 (92.7 ± 1.2)	NE
mtB-M	188	179 (95.2 ± 3.1)	47 (26.3 ± 2.7)*	19 (10.6 ± 4.2)

mtM-M: mouse embryos with mitochondria derived from another mouse. mtB-M: mouse embryos with bovine mitochondria. NE: Not examined.

^*^Column with an asterisk differs from non-manipulated embryos (P < 0.05.).

**Table 2:** Lethality of the mouse embryos harbouring bovine mitochondria at the early postimplantation stage.

	No. of recipients	No. of blastocysts transferred	No. of implantation sites(% ± SEM)	No. of viable foetuses(% ± SEM)
non-manipulated	4	38	24 (63.2 ± 9.2)	15 (39.5 ± 11.7)
mtM-M	3	30	22 (73.3 ± 3.3)	8 (26.7 ± 3.3)
mtB-M	7	51	0 (0.0 ± 0.0)	0 (0.0 ± 0.0)

mtM-M: mouse embryos with mitochondria derived from another mouse. mtB-M: mouse embryos with bovine mitochondria.
